# A Case of Anaplasty of the Palate

**Published:** 1867-10

**Authors:** E. Franklin Coates

**Affiliations:** Mystic Bridge, Conn.


### ARTICLE VI.

### *A Case of Anaplasty of the Palate.*

By E. Franklin Coates, M.D., Mystic Bridge, Conn.

The operation for the cure of congenital cleft palate, though not new, is not very common, for "failure after failure has been the common result, except in some rare instances and most favorable subjects."

The patient, John Tufts, aged 24 years, was born with the *uvula* in two parts, the cleft extending a little more than one inch upward into the *velum palati.* I found on each side of the palatine arch muscular structure enough, so that with forceps the parts could be drawn together ; therefore decided to operate. July 12th, '67. I held the uvula of one side and its pillar with forceps, and with angular scissors commenced by cutting the mucuous membrane from its side. I then seized this membrane, holding it (instead of both it and the velum) with the forceps, and with a small scalpel (instead of the blunt pointed bistoury usually recommended,) I was enabled to skin a larger surface of the membrane from the edge than I could have done by holding the muscular structure also. I was careful to hold the edge of the knife towards the velum so as not to cut away the detached membrane, but leave it entire to the upper part of the slit. When this was done I left the membrane hanging, and then operated on the other side in the same way, cutting up to where I left off on the opposite side, removing the whole membrane of both sides of the opening in one piece.

I then held the velum with forceps, and with a small, rather short, crooked needle, held in bent needle forceps, I took the first suture near the upper part, being careful that the needle did not penetrate the nasal surface, and that the suture passed through the centre of the denuded edge. The hold was broad, being about three-sixteenths of an inch on each side, The suture used was common machine silk. In this way I entered three threads with their ends hanging out at each corner of the mouth. I then inserted the fourth at a suspicious place, so as to be sure and hold the parts together.

I then knotted the sutures in the order of their insertion, but found the common surgeon's knot would slip, therefore made three turns, which answered my purpose better. Now, after the sutures were all tied and their ends cut, in order to give additional security, another broad, deep suture was introduced through the entire velum into the nasal fossa, and brought out through the velum of the opposite side, so as to encircle the cut. This was firmly tied and the operation was complete.

July 15th. Three days after the operation, one suture becoming loosened, was removed, after which, as they became loose, the sutures were removed daily, until July 18th, in six days, the last was removed, and the parts were united together ; and now, July 26th, in two weeks, the inflammation has gone, and the parts appear as sound as though nature had formed them in their present position.

After the operation, the patient was directed to live on liquid food, milk, beef tea, etc., and to avoid everything that could in the least irritate the throat.

As the parts to be brought together are extremely thin, it is necessary that we dissect the mucous membrane in such a manner as to expose as much raw surface as possible. I believe the success of this case depended upon holding the membrane alone in the forceps and the use of the scalpel, with the point of which I was enabled to denude a larger surface than I could have done with any other instrument. By leaving the dissected membrane unbroken from the point of the uvula of one side up and down the entire sides of the triangle to the point of the uvula of the opposite side, I was sure that every part of the cut edge was thoroughly denuded, without which the operation would be fruitless, or but a partial success.

I would not attempt to operate unless the patient could so far control himself as to let me draw the parts together without serious flinching, for the operation is a delicate one, the parts to be brought together, especially at the upper part, being very like the shaved edges of two bits of calf skin, so far as surface is concerned, and not only requires a mechanical eye and dexterous hand, but a determined and obedient patient.—*New York Medical Journal.*

